# Identification of a novel isolated 4q35.2 microdeletion in a Chinese pediatric patient using chromosomal microarray analysis: a case report and literature review

**DOI:** 10.1186/s13039-023-00651-3

**Published:** 2023-08-02

**Authors:** Jianlong Zhuang, Shufen Liu, Xinying Chen, Yuying Jiang, Chunnuan Chen

**Affiliations:** 1 Prenatal Diagnosis Center, Quanzhou Women’s and Children’s Hospital, Quanzhou, 362000 Fujian Province People’s Republic of China; 2 Department of Neurology, The Second Affiliated Hospital of Fujian Medical University, Quanzhou, 362000 Fujian People’s Republic of China

**Keywords:** 4q35.2 microdeletion, SNP array, Whole exome sequencing, Genetic etiology, Congenital anomalies

## Abstract

**Background:**

Isolated terminal 4q35.2 microdeletion is an extremely rare copy number variant affecting people all over the world. To date, researchers still have controversial opinions and results on its pathogenicity. Here, we aim to present a Chinese pediatric patient with terminal 4q35.2 microdeletion and use this case to clarify the underlying genotype–phenotype correlation.

**Methods:**

A 17-year-old boy from Quanzhou, South China, was recruited as the main subject in this study. Karyotype and single-nucleotide polymorphism (SNP) based microarray analysis were carried out to detect chromosomal abnormalities and copy number variants in this family. Trio whole exome sequencing (Trio-WES) was performed to investigate the potential pathogenic variant in this family.

**Results:**

During observation, we identified abnormal clinical phenotypes including upper eyelid ptosis, motor developmental delay, abnormal posturing, abnormality of coordination, attention deficit hyperactivity disorder, and involuntary movements in the patient. SNP array analysis results confirmed a case of 2.0 Mb 4q35.2 microdeletion and parental SNP array verification results indicated that the terminal 4q35.2 microdeletion was inherited from his mother. No copy number variants were detected in his father. In addition, the trio-WES results demonstrated none of pathogenic or likely pathogenic variants in the patient.

**Conclusions:**

This study brings a novel analysis of a case of 2.0 Mb terminal 4q35.2 microdeletion affecting a Chinese individual. In addition, additional clinical symptoms such as upper eyelid ptosis and involuntary movements were first reported to affect a patient with terminal 4q35.2 microdeletion, which may broaden the phenotype spectrum of the condition.

## Introduction

In China, approximately 5.6% of the population suffers congenital defects after birth, with chromosomal abnormalities being the most common reason for birth anomalies [1]. Karyotyping for long was considered as a traditional chromosomal abnormality detection tool with a low resolution. At present, chromosomal microarray analysis (CMA) can rapidly and effectively detect most of chromosomal abnormalities including copy number variants (CNVs) and uniparental diploid/triploid and has been proposed to be the first-tier genetic diagnostic tool for patients with developmental disabilities or congenital diseases [2, 3]. Moreover, CMA technology has been increasingly utilized in patients with developmental delay/intellectual disability (DD/ID), autism spectrum disorders, and multiple congenital anomalies (MCA) and indicated a higher rate of chromosomal defects in patients diagnosed with DD/ID and MCA [4, 5]. In the clinical practice, whole exome sequencing (WES) technology has been recommended as a sharp tool to investigate the additional sequence variants in patients with unexplained CNVs [6, 7].

Terminal chromosome 4q35.2 deletion is an uncommon event and typically manifests as developmental delay, craniofacial anomalies, intellectual disability and other dysmorphisms [8, 9]. Previous studies indicated that the 4q33 region may be the crucial affected region for 4q deletion syndrome and result in more severe clinical features, while, patients with a more distal region of 4q34q35 may have a mild dysmorphic phenotype and less impacting intellectual disabilities [10, 11]. The 4q35 microdeletion is rarer with few reports available and exhibits obvious phenotypic heterogeneity. However, researchers still have controversial opinions regarding its pathogenicity.

In this study, both CMA and WES technology were carried out to reveal the genetic etiology in a Chinese pediatric patient with clinical phenotypes including upper eyelid ptosis, motor developmental delay, abnormal posturing, coordination abnormalities, attention deficit hyperactivity disorder, and involuntary movements.

## Case presentation

The subject of this study is a male pediatric patient and also the first child in his family. During the pregnancy, threatened abortion occurred at 12 weeks, and he was born at 7 months, with a weight of 1900 g. A special fist-clenching posture was observed after birth according to his parents’ description. Subsequently, motor developmental delay was diagnosed at his age of 2, without obvious craniofacial malformation, except for a upper eyelid ptosis. Involuntary movements, inability to concentrate, abnormal running posture, and obvious motor retardation were presented at the age of 4 and the boy was subsequently motor developmental delay, abnormal posturing, coordination issues, attention deficit hyperactivity disorder, and involuntary movements were diagnosed. His EEG detection results came back normal and the Wechsler Intelligence Scale for Children results elicited an intelligence score of 73, which was at the borderline of ID. In addition, the parents described that learn disability was observed during his upbringing and claimed that he seldom has communicated with others. At present, the boy is 17 years old, with 173 cm in height and 64 kg in weight. The parents claimed that involuntary movements occur less frequently than in childhood, but still happen occasionally.

## Results

**Fig. 1:**
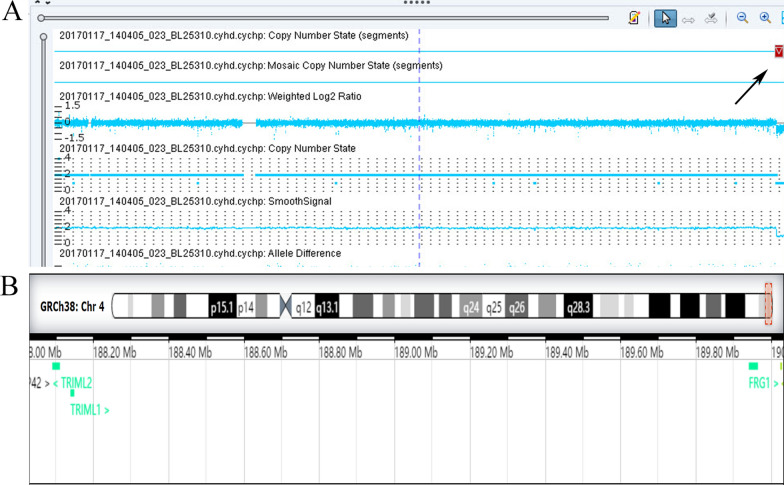
SNP array detection results in the patient of our study. **A** The SNP array analysis result demonstrates a 2.0 Mb deletion in 4q35.2 region (arr[GRCh37]4q35.2(188,952,176–190,957,473) × 1), the arrow indicates the deletion region. **B** In the deletion region, four protein code genes were covered, including *TRIML1, TRIML2, FRG1* and *FRG2*

Karyotype analysis demonstrated a normal male karyotype of 46, XY in the patient, and no chromosomal abnormalities were identified in his parents as well. SNP array detection demonstrated a 2.0 Mb deletion in the 4q35.2 region (arr[GRCh37]4q35.2(188,952,176–190,957,473) × 1) (Fig. 1), which contained two OMIM genes: *FRG1* (MIM 601278) and *FRG2* (MIM 609032). According to the standards and guidelines of American College of Medical Genetics (ACMG) and ClinGen, the 4q35.2 microdeletion was interpreted as variants of unknown significance (VOUS). In addition, parental SNP array verification results indicated that the CNVs was inherited from his mother.

The terminal 4q35.2 microdeletion region detected during our clinical experiments was not found in the DGV database. In addition, the 4q35.2 terminal region did not cover the haploinsufficiency dosage-sensitive gene or genome according to the ClinGen database. As delineated in the DECIPHER database, three different cases with smaller deletion regions were interpreted as pathogenic/likely pathogenic variants (DECIPHER ID: 286,230, pathogenic; 331,349, likely pathogenic; 331,286, likely pathogenic), while, a case with deletion fragment similar to our case was classified as likely benign variant (DECIPHER ID: 413,380).

To better understand our results, the clinical findings and molecular analysis of isolated terminal 4q35.2 microdeletion reported in other similar studies were properly reviewed and listed in Table 1. Furthermore, trio-WES was carried out to investigate the additional sequence variants in our patient. However, no pathogenic gene variants that are relevant to his clinical phenotypes were detected (Table 2). Additionally, seven variants were screened out; those being inherited from his parents and considerably relevant to partial clinical features affecting the patient. Among them, the NM_000292: c.1828A > G (p.T610A) variant in the *PHKA2* gene was associated with an X-linked recessive inheritance disease coming from his mother who has normal clinical features. In addition, further investigation of other male members in the family elicited that the variant in *PHKA2* was also identified in the patient’s uncle who also exhibit normal clinical phenotypes.

**Table 1:** Clinical findings and molecular genetic analysis in patients with isolated CNVs of distal 4q35.2 microdeletion (Literature review)

References	Age/Sex	Deletion	Size	Inheritance	Clinical features
Karaman et al. [12]	2years/F	4q35.2 (Homozygous)	204.947 kb	Parental	Various facial dysmorphic features, developmental delay, postnatal growth retardation, intellectual disability, and seizures
Cuturilo et al. [13]	2years2months/M	4q34.1q35.2	17.4 Mb	De novo	Tetralogy of Fallot, right aortic arch and facial dysmorphism
Pickard et al. [14]	NA	4q35.2	3.0 Mb	NA	Comorbid schizoaffective disorder and mild intellectual disability
Rossi et al. [15]	17years/F	4q34.1q35.2	16.435 Mb	De novo	Developmental delay, learning disability, ADHD, primary amenorrhea, myopia, and minor facial dysmorphic features, clinodactyly of the left and right fifth toes
Youngs et al. [16]	12years/M	4q35.2	1.2 Mb	NA	Facial dysmorphic features, fifth finger clinodactyly, hyperflexible, toe, malalignment, ADHD, aggressive behavior
Balikova et al. [17]	7years/M	4q35.2	1.3 Mb	Maternal	Mild intellectual disability
Fu et al. [18]	Fetus	4q35.2	1.58 Mb	NA	Bilateral multicystic dysplastic kidneys
Xiao et al. [19]	Fetus	4q35.1q35.2	4.5 Mb	Maternal	Fetal growth restriction without significant abnormalities
Riccardi et al. [11]	39years/M	4q35.2	269.34kb	NA	Crohn’s disease
Present study	17years/M	4q35.2	2.0 Mb	Maternal	Motor developmental delay, abnormal posturing, learning disability, ADHD, involuntary movements, and upper eyelid ptosis

*NA* Not available; *F* Female; *M* Male; *ADHD* Attention deficit hyperactivity disorder

**Table 2:** Variants identified by whole exome sequencing in our subject

Gene	RefGene	Variants	Mode of inheritance	Genotype	Asian frequency	ACMG guidelines	Original
*PHKA2*	NM_000292	c.1828A > G p.T610A	XLR	hem	–	VOUS	Maternal
*CDH2*	NM_001792	c.1471G > C p.V491L	AD	het	0.0002	VOUS	Maternal
*CIC*	NM_001386298	c.6058C > T p.P2020S	AD	het	0.0002	VOUS	Paternal
*KAT6A*	NM_006766	c.4825A > G p.M1609V	AD	het	0.0003	VOUS	Paternal
*NALCN*	NM_052867	c.1587C > G p.C529W	AR,AD	het	0.0001	VOUS	Maternal
*CTC1*	NM_025099	c.2386-1G > A	AR	het	0.0007	LP	Maternal
*MANBA*	NM_005908	c.280C > A p.Q94K	AR	het	0.0011	VOUS	Maternal

*XLR* X-linked recessive; *AD* Autosomal dominant; *AR* Autosomal recessive; *VOUS* Variants of unknown significance; *LP* Likely pathogenic; *hem* Hemizygote; *het* Heterozygote

## Discussion

At present, the genotype–phenotype correlation of 4q35.2 microdeletion has not been fully understood, despite the many efforts inside the scholarly community. Chromosome 4q35.2 microdeletion is an uncommon chromosomal abnormality with obvious phenotypic heterogeneity. As listed in Table 1, we reviewed 10 cases of isolated 4q35.2 microdeletion and identified that in three cases the gene was inherited from the mother directly and two cases were de novo. In addition, evident phenotypic heterogeneity was observed in the patients, with the most common affected features being facial dysmorphic features (5/10), followed by intellectual disability (3/10), developmental delay (3/10), attention deficit hyperactivity disorder (ADHD) (3/10), learning disabilities (2/10) and fifth finger or toe clinodactyly (2/10).

Our subject underwent an assessment of intellectual disability and obtained a borderline scores of 73, which may possibly affect his mental development. A similar study conducted by Riccardi et al. [11] presented the case of a patient who had a terminal deletion of 4q35.2 and also had a borderline IQ score, with duplication of Xp22.11 and deletion of 13q34. It is noteworthy that the clinical phenotypes of developmental delay, ADHD, and learning disabilities observed in our patient have been widely reported in cases with 4q35.2 microdeletion detected in other research [12, 15, 16].

To date, researchers still have controversial opinions on the pathogenicity of small deletions on the long arm of distal chromosome 4. A recent prenatal study [19] suggested that the 4q subtelomeric region deletion is a familial variation and indicated that the 4q35.1q35.2 region single-copy deletion would not cause obvious congenital defects or intellectual disability. On the other hand, fetal growth restriction was observed in the fetal period (in the studies cases), so it cannot be ruled out that intellectual disability may occur during in childhood and after birth. In addition, another similar study conducted by Akbas et al. [20] demonstrated a case of 2.449Mb terminal 4q35.2 microdeletion in a fetus with growth retardation, also associated with 4p16.3 deletion (size 130 kb without covering the Wolf Hirschhorn critical region). Another related study [21] elicited a 5.75 Mb interstitial deletion in the 4q35.1q35.2 region in the mother and her two daughters who had no remarkable phenotypic defects. In our case, the 4q35.2 microdeletion in the patient was inherited from his mother (who did not have abnormal clinical features), which indicates incomplete penetrance.

In the present study, the patient harbour a terminal 4q35.2 deletion that covering *FRG1* and *FRG2* genes. *FRG1* located closely to an integral number of tandem 3.3-kb repeats, termed D4Z4, which was commonly deleted in most patients with facioscapulohumeral muscular dystrophy (FSHD). FSHD is associated with contraction of the D4Z4 macro satellite repeat, of the unaffected individuals, the D4Z4 array consists of 11 to 150 repeat units, while, the FSHD patients have D4Z4 repeat units from 1 to 10, due to over-expression of FRG1 [22, 23]. FSHD is a progressive skeletal muscle disorder with a highly variable phenotype and incomplete penetrance, typical manifest as striking asymmetry of muscle involvement from side to side and sparing of bulbar extraocular and respiratory muscles [24]. In addition, a previous study indicate that *FRG1* appears to be the candidate gene for penetrance and severity of FSHD [11]. Moreover, a previous study [25] suggested that the penetrance of the FSHD was significantly greater for males (95%) than for females (69%). In our case study, more work need to be done to investigate the repeat units of D4Z4 to determine whether the FSHD would be diagnosed in the patient.

WES technology was used to investigate the additional variant in our patient. No pathogenic variant was detected by WES. However, a NM_000292: c.1828A > G (p.T610A) variant in *PHKA2* gene was observed in the patient and associated with an X-linked recessive inheritance disease coming from the mother side. *PHKA2* gene mutations can often cause deficiencies of hepatic phosphorylase kinase activity and result in glycogen storage disease type IXa, with typical clinical symptoms involving combinations of hypoglycaemia, hepatosplenomegaly, short stature, hepatopathy, weakness, fatigue, and motor delay [26, 27]. However, our patient did not have most of these relevant features, except for motor developmental delay. In addition, the *PHKA2* gene variant was also identified in the patient’s uncle, who manifest normal clinical phenotypes. Thus, we believe that the c.1828A > G (p.T610A) variant in the *PHKA2* gene identified in our patient may not responsible for his clinical anomalies.

To our best knowledge, there have been no reports about patients with 4q35.2 microdeletion that led to the clinical features of involuntary movements and upper eyelid ptosis. We believe both of the involuntary movements and upper eyelid ptosis in the patient may ascribe to the 4q35.2 microdeletion.

In conclusion, our study is a novel analysis of a case of 2.0 Mb 4q35.2 microdeletion in a Chinese pediatric patient. Both of the clinical symptoms of upper eyelid ptosis and involuntary movements were first identified in a patient with 4q35.2 microdeletion, which may broadened the phenotype spectrum of this genetic syndrome.

## Data Availability

The datasets used and analyzed during the current study are available from the corresponding author on reasonable request.
